# Experimental Study on Enhanced Oil Recovery Effect of Profile Control System-Assisted Steam Flooding

**DOI:** 10.3390/polym15234524

**Published:** 2023-11-24

**Authors:** Long Dong, Fajun Zhao, Huili Zhang, Yongjian Liu, Qingyu Huang, Da Liu, Siqi Guo, Fankun Meng

**Affiliations:** 1 Heilongjiang Provincial Key Laboratory of Oilfield Applied Chemistry and Technology, School of Chemical Engineering, Daqing Normal University, Daqing 163712, China; 2 Key Laboratory of Entered Oil Recovery of Education Ministry, Northeast Petroleum University, Daqing 163318, China

**Keywords:** steam flooding, foam, profile control, enhance oil recovery, high temperature

## Abstract

Steam flooding is an effective development method for heavy oil reservoirs, and the steam flooding assisted by the profile control system can plug the dominant channels and further improve the recovery factor. High-temperature-resistant foam as a profile control system is a hot research topic, and the key lies in the optimal design of the foam system. In this paper, lignin was modified by sulfonation to obtain a high-temperature-resistant modified lignin named CRF; the foaming agent CX-5 was confirmed to have good high-temperature foaming ability by reducing the surface tension; the formula of the profile control system (A compound system of CRF and CX-5, abbreviated as PCS) and the best application parameters were optimized by the foam resistance factor. Finally, the effect of PCS-assisted steam flooding in enhanced oil recovery was evaluated by single sand packing tube flooding, three parallel tube flooding, and large-scale sand packing model flooding experiments. The results show that CX-5 has a good high-temperature foaming performance; the foam volume can reach more than 180 mL at 300 °C, and the half-life is more than 300 s. The optimal PCS formulation is 0.3 wt% CRF as an oil displacement agent + 0.5 wt% CX-5 as a foaming agent. The optimal gas–liquid ratio range is 1:2 to 2:1, and the high pressure and permeability are more conducive to the generation and stability of the foam. Compared with steam flooding, PCS-assisted steam flooding can improve oil recovery by 9% and 7.9% at 200 °C and 270 °C, respectively. PCS can effectively improve the heterogeneity of the reservoir, and increase the oil recovery of the three-parallel tube flooding experiment by 28.7%. Finally, the displacement results of the sand-packing model with large dimensions show that PCS can also expand the swept volume of the homogeneous model, but the effect is 9.46% worse than that of the heterogeneous model.

## 1. Introduction

Oil and natural gas will still be the main energy sources in the world in the next few decades. Developing unconventional oil and gas reservoirs will guarantee stable oil and gas production. Heavy oil reservoirs have a very unfavorable water–oil mobility ratio, and steam flooding is a favorable means for their efficient development [1,2,3,4,5]. However, with the continuous injection of steam, channeling is intensified, influenced by factors such as well spacing, reservoir heterogeneity, crude oil properties, gravity separation, reservoir pressure and temperature, relative permeability, steam quality, mobility ratio, and fluid saturation in the formation [6,7]. Control of steam channeling to expand the swept volume is an important guarantee for the heavy oil reservoir to expand the swept volume further and improve oil recovery.

Currently, a common method to solve the problem of gravity overload and the steam channeling of steam flooding is profile control agent-assisted steam flooding. The profile control system is injected into the reservoir with steam to plug the flow channels so that the steam injected into the reservoir will enter the unswept area [8,9,10]. The research proposed by Stanford University [11] comprehensively considered various chemical additives related to mobility control and considered applying the profile control technology in the steam flooding process an effective method [12]. The commonly used profile control systems include association polymer [13], microgel [14,15,16,17], water-assisted gas flooding [18,19], and foam [20].

Foam as a profile control system [21,22] has the mechanism of reducing steam mobility, increasing swept coefficient, and improving oil washing efficiency, which can improve the ultimate oil recovery of the reservoir. Meanwhile, the foam will not permanently block the oil layer because of the good selectivity of its plugging property. In the early 1970s, Needham [23] and others invented the steam foam flooding technology. Since then, American Shell Oil Company, CLD Group Company, Stanford University Petroleum Research Institute, and Getty Oil Company have conducted in-depth laboratory research on steam foam flooding technology, and conducted field test research in the Midway-Sunset and North Kern Front oilfields, and have achieved initial success [24]. The U.S. Department of Energy signed cost-sharing research contracts with Petio-Lewis Corporation, CLD, and SUPRI from 1979 to 1980, and entrusted Chemical Oil Recovery Company as the steam foam flooding technology manager from the laboratory to the field [25,26,27,28,29]. Surfactant/foam injected into the formation, together with steam, will preferentially enter the formation with steam overburden and channeling and temporarily plug the channeling channels [23,30], diverting the steam direction to expand the swept volume [31,32]. Borchard [33], Ettinger et al. [34], and Schramm et al. [35] carried out a large number of experimental studies, respectively, and concluded that the use of foam plugging in the steam flooding process is effective.

Although the above laboratory studies have shown that foaming agents can be used as plugging agents for steam flooding, the high price and poor temperature resistance of the foaming system limit its application in the oilfields [36,37,38,39]. Therefore, the development of a foam system with excellent foamability and high-temperature stability is the key to the study of foam profile control technology [40]. The injection of a suitable concentration of lignosulfonate solution into the formation can generate a gel and plug the channeling channel. Wang et al. [41] pointed out that the glue-forming liquid prepared with lignin extracted from paper-making waste liquid as the main material can form a good gel under the conditions of pH > 9 and temperature of 50~150 °C. Therefore, this paper considers the use of cheap modified lignin to improve the temperature resistance and the foam stability of the composite system.

This paper conducts research on the design of high-temperature steam flooding assisted by a profile control system, using modified lignin to improve the temperature resistance and foam performance of the system. Firstly, temperature-resistant modified lignin (CRF) from industrial wastewater was prepared via chain scission and sulfonation reactions, and then its properties were characterized by means of infrared spectroscopy, ultraviolet spectroscopy, and a TGA test. Meanwhile, the high-temperature foaming performance, foam stability, and ability to reduce the surface tension of the solution were evaluated for the foaming agent CX-5. Through the sand pack resistance factor experiment, the optimal formulation of the profile control system (PCS, including CX-5) was optimized. Finally, a single sand pack flooding efficiency experiment, a parallel flooding experiment with three sand-packing tubes, and a large-size core flooding experiment were carried out to clarify the EOR effect of PCS-assist steam flooding. The research contained in this paper can provide an experimental basis and data supporting the optimization of the on-site steam flooding profile control system and the optimal design of the injection parameters.

## 2. Material and Method

### 2.1. Materials

**Agent:** Pure lignin (The molecular weight is 3.2 × 10^5^ Da) is obtained by acid precipitation and purification of low-concentration reed papermaking black liquor from Daqing Haida Paper Co., Ltd. (Daqing, China). Brine is prepared based on the composition of formation water in Daqing Oilfield. It contained 2359.8 ppm Na^+^ + K^+^, 4278.2 ppm HCO_3_^−^, 1018.6 ppm Cl^−^, 175.2 ppm CO_3_^2−^, 110.7 ppm SO_4_^2−^, 35.4 ppm Ca^2+^ and 22.1 ppm Mg^2+^. High temperature resistant foaming agent CX-5 (the molecular weight is 7.4 × 10^3^ Da) is an anionic sulfonate surfactant with an effective concentration of 15%, which is provided by Daqing Petroleum Refinery (Daqing, China); the composite solution prepared by modified lignin (CRF) and CX-5 according to a certain concentration is the profile control system (PCS). Sulfuric acid, hydrochloric acid, ethanol, NaOH, formaldehyde, nonylphenol, sodium sulfite, hydrogen peroxide, etc., were purchased from Shanghai Aladdin Company (Shanghai, China), and were analytically pure.

**Crude oil and brine:** The deionized water was prepared by the ultra-pure water filter of Chengdu Youpu Company (Chengdu, China). According to the formation water formula, inorganic salt was added to prepare simulated formation water, and its salinity was 8000 mg/L. The crude oil is degassed and dehydrated crude oil from Liaohe Oilfield (Panjin, China), with a viscosity of 63,670 cP at room temperature, and reduced to 10 cP when heated to 200 °C.

**Sand packing model:** The model is 60 cm long, 2.5 cm in diameter, and filled with 50–220 mesh quartz sand. The permeability can be adjusted by designing the proportion of quartz sand with different mesh numbers, and it can be controlled between 1.0 D–15 D.

**Rectangular sand packing model:** The model was welded with stainless steel, and its dimensions are as follows: length × width × height = 1200 mm × 40 mm × 120 mm. There is a heating and insulating layer outside the core model; the initial temperature of the core can be set, and experiments can be carried out under thermal insulation conditions. The quartz sand used has 60 mesh, 70~140 mesh, and 270 mesh. The model is divided into the homogeneous model (place the sand packing model vertically, compact it evenly when filling, and try to ensure that the permeability is the same everywhere); and the heterogeneous model (place the sand packing model vertically, place a layer of the screen in the middle of the model, and fill the two sides of the screen with quartz sand of different meshes to form different permeability layers). After filling, the model is placed horizontally, the upper layer has a high permeability and the lower layer has a low permeability, so as to simulate the heterogeneity of the formation.

### 2.2. Lignin Modification Method

(1) Oxidative chain scission of lignin. A certain amount of lignin was dissolved in NaOH solution with pH = 10 and then reacted with O_2_ and 20% H_2_O_2_ in a 0.3 L autoclave. The reaction pressure was controlled at 0.4 Mpa, the reaction temperature was 70 °C, and the reaction time was 1.5 h. After the reaction, the product was concentrated and dried for analysis. (2) Synthesis of modified lignin surfactant (CRF). A total of 6 g of sodium sulfite and 9 g of formaldehyde were added to the above-purified sample, and 3 g of nonylphenol was weighed and dissolved in an organic solvent, then added to the autoclave, and then heated to 70 °C. After the reaction was completed and cooled to room temperature, the reactant was filtered and the precipitate was collected, vacuum-dried at 50 °C, and finally, the modified lignin solid brown powder was obtained.

### 2.3. Modified Lignin Properties

The sample was pressed into a film, and its infrared spectrum (wave number 4000–450 cm^−1^) was measured with a Fourier transform infrared spectrometer (Spectrum 400, Bruker, Billerica, MA, USA, potassium bromide tablet); ethanol was used as a solvent, and an ultraviolet-visible absorption spectrometer (UV-540, Shanghai Metash Instruments Co, Ltd, Shanghai, China) was used to determine the UV spectrum of the sample (wavelength 190–380 nm); the content of carbon, hydrogen, and oxygen elements in the lignin was analyzed with an elemental analyzer (EA-300 series, Keyence, Shanghai, China); a thermogravimetric analyzer (TA Q50, Dupont, Wilmington, DE, USA) was used to perform a thermogravimetric test on the CRF (6.523 mg of the sample was weighed, with high purity N_2_ used as the carrier gas. The heating rate of the thermogravimeter was 15 °C/min, and the temperature was heated from room temperature to 700 °C).

### 2.4. Foaming Performance of the Foaming Agent

#### 2.4.1. Determination of Foam Stability by Bubbling Method

**Determination of foam stability at room temperature and pressure:** Put 20.0 mL of foaming agent solution into the container of the high-temperature and high-pressure foaming device (300 °C, 50 MPa, Huabao, Yangzhou, China), and blow 140.0 mL of N_2_ at a certain speed. The change in foam height reflects the dynamic balance change in foam formation and collapse, so it is a comprehensive reflection of foam stability and foaming ability. Measure the change in the foam volume with time after the bubbling is stopped, draw the foam decay curve, and obtain the foam half-life t_0_. In the experiment, a circulating water bath was used to control the temperature of the system.

**Determination of high-temperature and high-pressure foam stability:** Put 20.0 mL of foaming agent solution into the container of the high-temperature and high-pressure foaming device, adjust the confining pressure of the system with N_2_, start the incubator to heat up to the predetermined temperature, and inject 140.0 mL at a certain speed after 1 h of N_2_ at a constant-temperature (condition of temperature and pressure in the container), measure the change in the foam volume with time after the bubbling is stopped, draw the foam decay curve, and obtain the foam half-life t_1_.

#### 2.4.2. Determination of Surface Tension of Foaming Agents

CX-5 solutions with concentrations of 0.05 wt%, 0.1 wt%, 0.3 wt%, and 0.5 wt% were prepared, and the surface tension was measured by the hanging ring method (CSI-356, Sincerity, Kansas, OK, USA) at 20 °C, and the surface tension was measured by the pendant drop method (Q2000, Hangzhou, China) at high temperature and high pressure, at 200 °C and 300 °C. At the same time, the air was injected into the solution to be tested through a syringe, and the change in the surface tension value within 1000 s was recorded to obtain the dynamic surface tension change curve of CX-5.

### 2.5. Resistance Factor of Profile Control System

**Figure 1:**
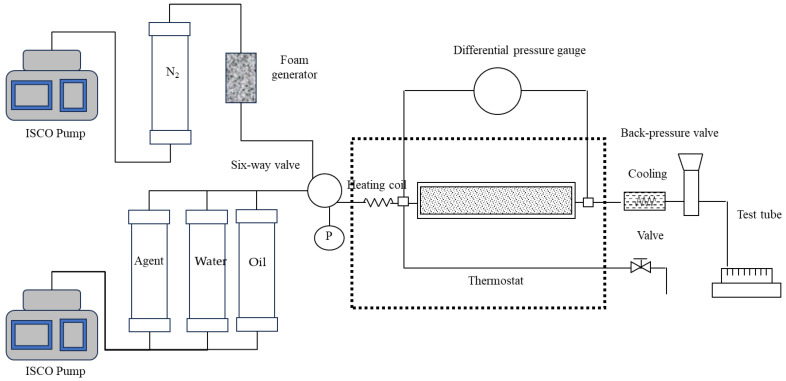
Schematic diagram of simulated experiment process in nitrogen foam steam flooding.

After it is clear that the foaming agent can generate stable foam, it is necessary to evaluate the actual plugging ability of the foam in the core to meet the plugging ability of the steam channeling layer. The plugging performance of the foam is usually evaluated by the resistance factor, calculated during the core flooding experiment. The foaming agent solution and air are injected simultaneously by the ISCO pump (ISCO D, 0.00001–408 mL/min, Teledyne ISCO, Lincoln, NE, USA), and the two are mixed at the inlet of the sand packing pipe. The pressure difference between the inlet and outlet of the core is measured by the pressure sensor, and the resistance factor is calculated. The specific experimental process is as follows:

(1) Design and make sand packing models of artificial quartz sand with different particle sizes to meet the requirements of the specific experimental plan for porosity and permeability. (2) After the sand pack is evacuated, the simulated formation water is saturated, and the porosity is measured. (3) Connect the experimental process according to the experimental flow chart as shown in Figure 1, and test the leakage at a pressure of 10.0 Mpa. (4) The constant temperature is 24 h, and the core flooding experiment is ready. (5) To determine the basic pressure difference P_0_, set the back pressure slightly lower than the saturated vapor pressure at this temperature, turn on the feed water pump to inject water, and turn on the air feed pump (set the displacement of the feed water pump and the air feed pump according to a certain gas–liquid ratio). Then, open the bypass valve to allow the steam and air mixture to pass through the bypass, and then close the bypass valve. When the pressure at the inlet of the sand packing pipe is slightly lower than the saturated vapor pressure at the experimental temperature, open the core inlet valve, and then open the core outlet valve to carry out the flow experiment. When the differential pressure reaches a stable level, record the basic pressure difference at both ends of the core model at this time. (6) To determine the working pressure difference P_1_, replace the water with the displacement agent solution, and carry out the experiment according to the same conditions and operating steps as (5). When the pressure difference across the model reaches a steady state, record the working pressure difference across the core model. (7) Calculate the foam resistance factor, that is, the ratio of the working pressure difference to the base pressure difference. (8) Repeat steps (1)–(7) by changing the experimental parameters and conditions. The specific experimental scheme is shown in Table 1.

### 2.6. Improved Oil Recovery Effect of Foam Assistant Steam Flooding

#### 2.6.1. Single-Tube Model Experiment

Firstly, the foam-assisted steam flooding efficiency experiment was carried out using a single sand packing model. The experimental flow chart is shown in Figure 1. The experimental steps are as follows: Step (1)–Step (3) are the same as those in Section 2.5. (4) Raise the incubator to the set temperature, keep at a constant temperature for 3 h, and use 3 to 5 times the pore volume of crude oil to displace the water in the core at a rate of 0.5 mL/min so that the oil saturation in the core meets the experimental design requirements. (5) For the steam + PCS flooding experiments, set the incubator at 270 °C for 5 h at a constant temperature, make the back pressure slightly lower than the saturated vapor pressure at this temperature, turn on the pump and inject water (or chemicals) and air into the foam generator according to the designed gas–liquid ratio. Then, open the bypass valve to let the injected mixed fluid pass through the bypass, so that the coil is completely filled with the mixed fluid, and then close the bypass valve. When the inlet pressure of the sand pack is slightly lower than the saturated vapor pressure at the experimental temperature, open the inlet valve and outlet valve of the sand pack, and then start the displacement experiment. (6) The amount of oil and water produced is calculated by solvent extraction distillation method. A total of 4 sets of experiments were carried out: (1) steam flooding; (2) steam + surfactant (CRF); (3) steam + CX-5 + air; (4) steam + PCS + air. During the injection process, the total injection speed was controlled to remain unchanged at 6 mL/min. If the air was injected, the gas–liquid ratio was 1:1, and the back pressure was 4.5 MPa.

#### 2.6.2. Three Parallel Sand Packing Model Displacement Experiment

The heterogeneity of the reservoir is simulated by the parallel sand packing tube model, and the effect of the control and flooding system on the channeling phenomenon during steam flooding and the effect of enhancing oil recovery are studied. The experimental steps are the same as those in Section 2.6.1, only the single sand packing model is replaced with a three-parallel sand packing model. The permeabilities of the three sand packing tubes are set to 1D, 4D, and 10D, respectively, and the model permeability gradient is 10. Four sets of experiments were also carried out: (1) steam flooding; (2) steam + surfactant (CRF); (3) steam + CX-5 + air; (4) steam + PCS + air.

#### 2.6.3. Large-Size Rectangular Sand Filling Mold Displacement Experiment

In order to further study the effect of the PCS in controlling steam overburden, blocking steam channeling channels, and expanding the swept volume of steam flooding, a large-scale sand-filled core insulating air foam steam flooding experiment was carried out. The flow chart of the experimental device and the experimental steps are the same as those in Section 2.6.1.

## 3. Result and Discussion

### 3.1. Modified Lignin Properties

#### 3.1.1. FT-IR Spectrum

**Figure 2:**
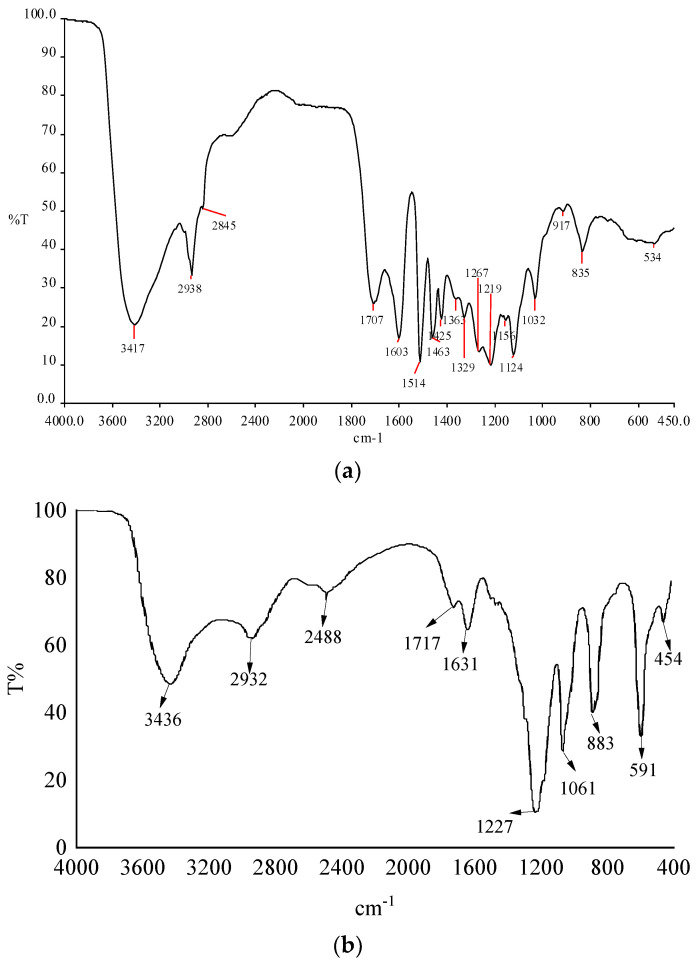
Comparison of infrared spectra of lignin (**a**) and CRF (**b**).

Figure 2 compares the infrared spectra of the lignin and modified lignin. Figure 2a has a strong hydroxyl absorption peak at 3417 cm^−1^, which is formed by the normal absorption peaks of hydroxyl at 3650 cm^−1^ and 3600 cm^−1^, which are shifted back and strengthened. This is due to the association of the hydroxyl hydrogen bonds on acid or base groups, indicating that lignin contains a large number of hydroxyl groups, and these hydroxyl groups form hydrogen bonds. Peaks at 1329 cm^−1^, 1219 cm^−1^, and 1124 cm^−1^ were mainly produced by syringyl. The peak at 1267 cm^−1^ belonged to the guaiac group in the lignin structural unit. From the comparison of the peak intensities of the two, it is shown that the content of the guaiac group in lignin is greater than that of the syringyl group. The peaks at 2845 cm^−1^ and 2938 cm^−1^ are due to the vibration of the methoxy groups, and the peaks at 3428 cm^−1^ are masked by the strong and broad absorption due to hydrogen bonding. The other peaks are consistent with the characteristic absorption peaks of chemical functional groups, see Table 2 for details.

Figure 2b still has a strong hydroxyl absorption peak at 3436 cm^−1^, indicating that there are still many hydroxyl groups that can continue to participate in the reaction after lignin sulfonation. The peak at 2932 cm^−1^ is due to the methoxy vibration, and at 1061 cm^−1^ is the coincident peak of the -SO_3_- antisymmetric and symmetric bond stretching vibration peaks. By comparing Figure 2a,b, it can be found that a large number of hydroxyl groups in lignin are involved in the oxidation reaction, and sulfonic acid is introduced, which proves the successful synthesis of modified lignin CRF.

#### 3.1.2. UV Spectral Comparison

**Figure 3:**
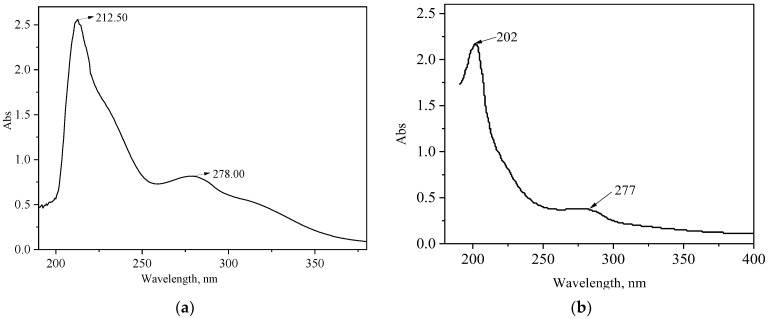
Comparison of UV spectra of lignin (**a**) and CRF (**b**).

Figure 3 compares the UV spectra of lignin and modified lignin. the lignin and CRF have absorption peaks around 212.50 nm and 202 nm, respectively, which are the absorption bands of conjugated olefinic bonds. A broad shoulder peak appears between 250 nm and 300 nm, indicating that there is a conjugated system in the molecule, which is the absorption band of the aromatic rings. The weak absorption peak around 278 nm indicates that the two have more symmetrical syringylbenzene ring structures. The UV spectrum shows that the CRF is still dominated by the aromatic structure of lignin, but sulfonate is introduced into the side chain through sulfonation. The benzene ring structure provides CRF with a strong temperature resistance.

#### 3.1.3. Elemental Analysis

From the elemental analysis in Table 3, it can be concluded that the molecular formula of CRF can be expressed as C_95_H_128_O_41_S_4_. The high C/H atomic ratio indicates that the lignin has a high content of benzene rings and a large proportion of oxygen, indicating that the lignin contains more hydroxyl groups, methoxy groups, and ether bonds.

**Figure 4:**
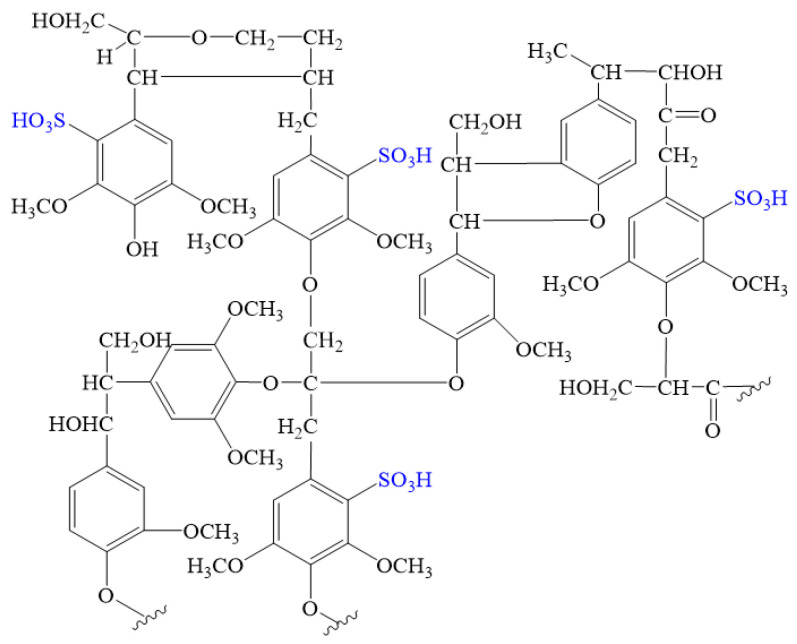
The simulated molecular formula of CRF.

It can be seen from the ultraviolet spectrum, infrared spectrum, and elements analysis that the modified lignin molecules mainly include guaiac groups and syringyl groups, of which syringyl groups are more than lignin groups. And the sulfonic acid group was introduced through oxidative modification, so the molecular chemical formula of CRF can be simulated, as shown in Figure 4.

#### 3.1.4. Thermogravimetric Analysis

**Figure 5:**
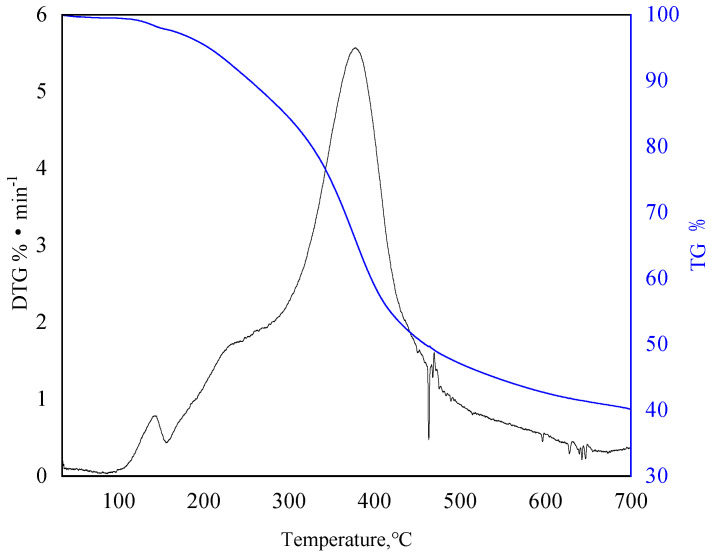
The TG curve and DTG curve of lignin (15 °C/min).

Figure 5 shows the thermogravimetric (TG) and differential thermogravimetric (DTG) curves of CRF at a heating rate of 15 °C/min. The obvious mass loss of CRF at 75~160 °C was caused by the desorption of free water, and the mass loss was 3.8%. The maximum mass loss rate was 0.75%/min, and the corresponding peak temperature was 140 °C. The main pyrolysis range of CRF is 235~415 °C, the mass loss is 58.58%, the maximum mass loss rate is 5.5%/min, and the corresponding peak temperature is 376.3 °C. The final yield of solid residue after pyrolysis was 39.8%, indicating that CRF has good temperature resistance in this temperature range.

### 3.2. Foaming Performance of CX-5

**Figure 6:**
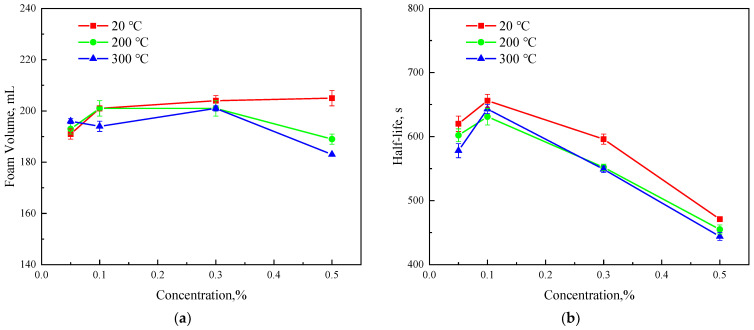
The influence of temperature on the foaming performance of CX-5. (**a**) Foam volume, (**b**) foam stability.

Figure 6 shows the foaming volume and half-life curves of CX-5 at the three temperatures 20 °C, 200 °C, and 300 °C. Figure 6a shows that at 20 °C, the foaming volume increases first and then tends to be stable with the increase in concentration. After the temperature was raised to 200 and 300 °C, the foaming volume of CX-5 slightly decreased, and the foaming volume with different CX-5 concentrations was similar and higher than 180 mL, showing good foaming performance. Figure 6b shows that the half-life of the foam decreases slightly after the temperature increases from 20 °C to 200 °C, but the half-life remains basically unchanged when the temperature continues to increase to 300 °C, showing good temperature resistance. When the concentration of CX-5 is 0.1 wt% at the three temperatures, the foam stability is the strongest; if the concentration of CX-5 continues to increase, the stability of the foam becomes worse. This is mainly because, when the concentration is higher than the critical micelle concentration, the enrichment of surfactant molecules on the surface of the solution reduces the liquid content and the stability of the foam. The half-life of CX-5 is more than 450 s as a whole, and it has good foam stability. Taking into account the foaming volume and half-life of the foam, the concentration range of CX-5 should be between 0.1 wt% and 0.3 wt%.

### 3.3. Surface Tension of CX-5

**Figure 7:**
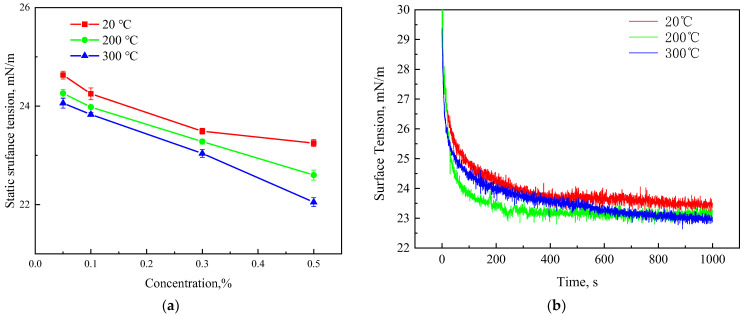
The influence of temperature on the surface tension of CX-5. (**a**) Static surface tension, (**b**) dynamic surface tension.

The foam system has a large specific surface area. The lower the surface tension, the more conducive to the stability of the foam, the less energy input from the outside is required, and the more conducive to the generation of foam. Therefore, surface tension is an important property of the foam system. Figure 7 shows the effect of temperature and concentration factors on surface tension.

Figure 7a shows that when the temperature is 20 °C, the surface tension of the solution first decreases and then tends to be stable with the increase in the concentration of CX-5; when the temperature increases to 200 °C and 300 °C, the surface tension of the solution continued to decrease with the increase in CX-5 concentration. At the same concentration, the surface tension of the solution decreases gradually with the increase in temperature. This is mainly because, as the temperature increases, the molecular thermal motion intensifies, which reduces the surface tension. Meanwhile, because CX-5 has good temperature resistance, it can still effectively reduce the interfacial tension at high temperatures. Figure 7b shows the change in the dynamic surface tension of the CX-5 solution. It can be found that the surface tension decreases rapidly during 0–200 s with the injection of gas, decreases slowly during 200–600 s, and stabilizes after 600 s. Figure 7 shows that the CX-5 solution has good surface activity, which can rapidly reduce the surface tension and maintain stability under high-temperature conditions, which is the basis for its good foaming performance.

### 3.4. Resistance Factor of the Foam

#### 3.4.1. Formulation Optimization of PCS

**Figure 8:**
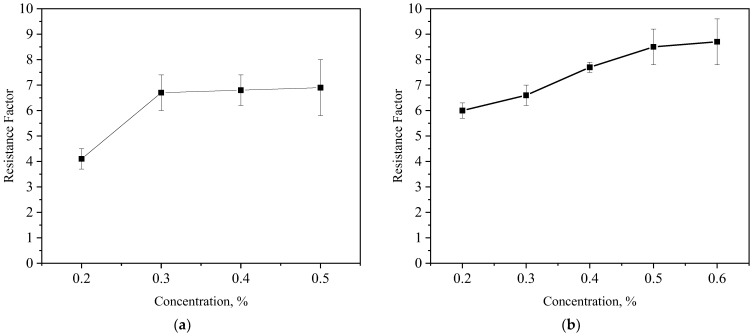
Resistance factor curves. (**a**) Different concentration of CRF (CX-5 is 0.3 wt%); (**b**) different concentration of CX-5 (CRF is 0.3 wt%).

Like polymer flooding, enhanced flow resistance is the basic EOR mechanism [42]. The two main agents of the CRF solution are the oil displacement agent CRF and foaming agent CX-5, and the recommended concentration of both is 0.2~0.5%, according to the static performance evaluation. In order to further clarify the optimal concentration of CRF and CX-5 in the PCS solution, resistance factor tests were carried out according to the experimental scheme in Table 1 (#1 and #2). The resistance factors of the different concentrations of CRF and CX-5 are shown in Figure 8.

It can be seen from Figure 8a that, when the concentration of CX-5 is constant (0.3 wt%), with the increase in CRF concentration, the resistance factor gradually increases, but when the concentration reaches 0.3 wt%, the increase decreases significantly. So, we set the concentration of CRF to 0.3 wt%. Figure 8b shows that when the CRF concentration is constant (0.3%), the resistance factor gradually increases with the increase in the CX-5 concentration, but, when the concentration reaches 0.5%, the increase decreases significantly. So, the concentration of CX-5 was set at 0.5 wt%. Finally, the formulation of the PCS solution for steam flooding in heavy oil reservoirs determined in this paper is 0.3 wt% CRF + 0.5 wt% CX-5.

#### 3.4.2. Influence of Gas–Liquid Ratio on Resistance Factor of PCS

Table 4 shows the calculation results of the resistance factor of the PCS solution when the gas–liquid ratio is 0:1, 1:2, 1:1, 3:2, and 2:1. When the gas-to-liquid ratio is too high or too low, it is unfavorable for the foam system to play a mobility control role in porous media. The gas–liquid ratio has a high resistance factor in the range of 1:2~2:1. Therefore, the gas–liquid ratio should be controlled as much as possible between 1:2~2:1 in the field construction.

#### 3.4.3. Influence of Temperature on Resistance Factor of PCS

Table 5 shows the resistance factors of PCS solution under the conditions of 120 °C, 150 °C, 200 °C, 270 °C, and 300 °C. As the temperature increases, the resistance factor decreases. When the temperature is 270 °C, the resistance factor is 8.4, and when the experimental temperature reaches 300 °C, the resistance factor is 5.8. In the process of steam injection, when the resistance factor is greater than 4, the injected flooding agent plays the role of diverting the steam. Therefore, when the temperature exceeds 270 °C, the injected PCS solution still has the effect of improving the displacement profile.

#### 3.4.4. Influence of Pressure on Resistance Factor of PCS

In order to evaluate the effect of pressure on the foam plugging ability, foam plugging experiments were carried out under the back pressure of 5.3 MPa and 8 MPa, respectively. The calculation results of the resistance factor are shown in Table 6. The increase in pressure can increase the stability of the foam, and, correspondingly, increase the plugging ability of the foam. As the pressure increases, the diameter of the foam becomes smaller, which is beneficial to the stability of the foam. After the pressure was increased from 5.3 MPa to 8 MPa, the resistance factor increased from 8.4 to 10.5.

#### 3.4.5. Influence of Permeability on Resistance Factor of PCS

The sand packs with the permeability of 1D, 4D, 10D, and 15D were used to evaluate the foam resistance factor, and the experimental results are shown in Table 7. The results show that with the increase in permeability, the resistance factor increases correspondingly, which shows that the foaming ability of air foam in the high-permeability region with large pores is higher than that in the low-permeability region with small pores. This is one of the important bases for using air foam to plug a high-permeability layer and improve the sweep coefficient.

### 3.5. Enhance Oil Recovery Effect of PCS Assistant Steam Flooding

#### 3.5.1. Single Sand Pack Flooding Experiments

**Figure 9:**
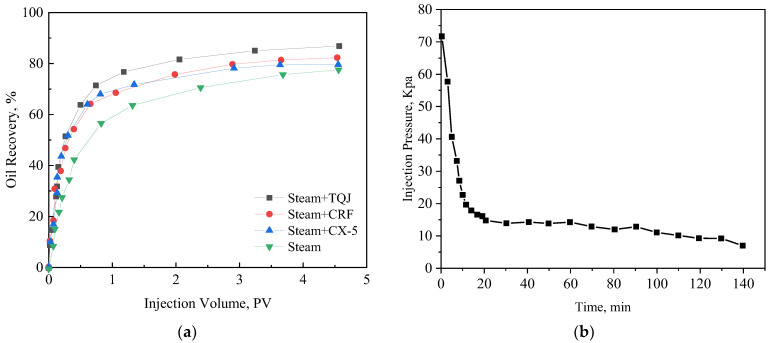
Oil displacement curves at 200 °C. (**a**) Oil displacement efficiency, and (**b**) the pressure differential curve of steam flooding.

**Figure 10:**
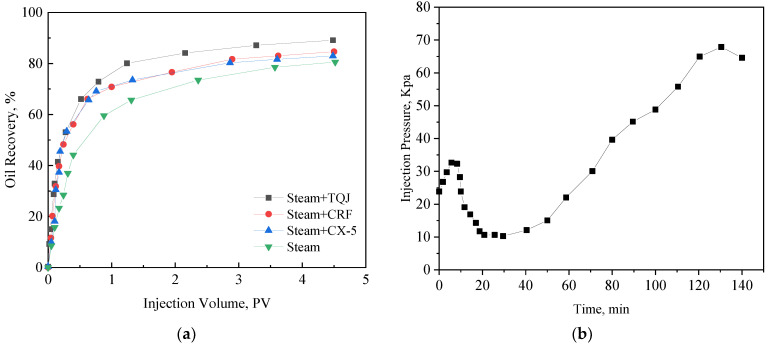
Oil displacement curves at 270 °C. (**a**) Oil displacement efficiency, (**b**) the pressure differential curve of TQJ assistant steam flooding.

Figure 9 and Figure 10 are the oil displacement characteristic curves of the four displacement modes at 200 °C and 270 °C, respectively. The steam flooding efficiency can be increased from 78% to 81.2% after the temperature is increased from 200 °C to 270 °C. Under the two temperature conditions, both CRF-assisted steam flooding and CX-5-assisted steam flooding can improve the oil displacement efficiency on the basis of steam flooding. Compared with thermal steam flooding, the oil displacement efficiency is increased by 3.5%, 1.7%, and 7.9%, respectively at 270 °C. When used alone, the oil displacement efficiency improvement of CRF is higher than that of CX-5. The combined use of the two can further improve the recovery factor by more than 6%, and the effect of PCS is good.

When the PCS solution is added to the hot water zone and the steam zone for displacement, the oil saturation in the sand pack gradually decreases as the displacement progresses, and the foam gradually plays a plugging role. From the pressure difference curve, it can be seen that the pressure difference between the two ends of the sand pack gradually increases in the middle and late stages of displacement (Figure 9b and Figure 10b), which is equivalent to a decrease in the steam mobility by up to eight times.

#### 3.5.2. Three Parallel Sand Packs Flooding Experiments

**Figure 11:**
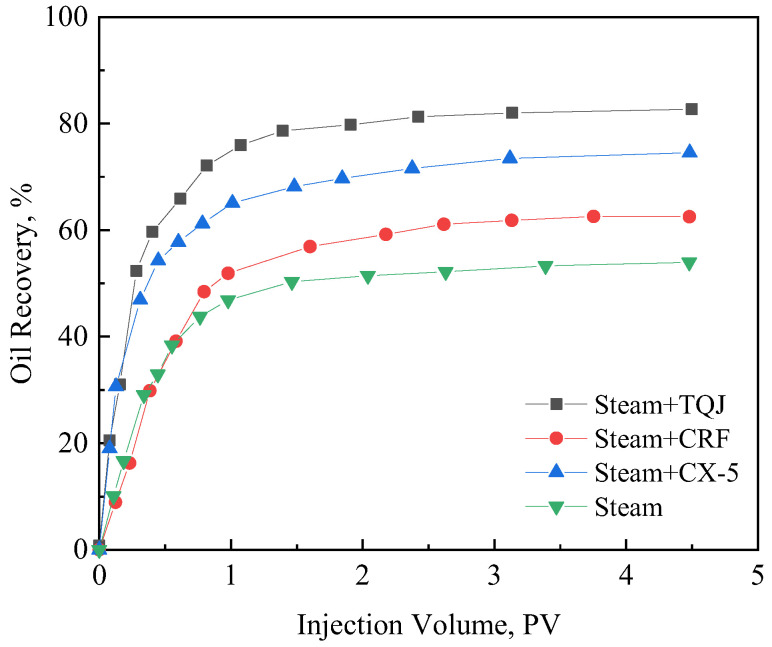
The curve of oil displacement efficiency and amount of injection PV in three tubes displacement experiments.

Figure 11 is the characteristic curve of the three parallel flooding experiments. During steam flooding, due to the serious formation heterogeneity, the sweep efficiency of the lower part of the oil layer is poor. When the flooding experiment is completed (the water cut of the produced liquid is 98%), the remaining oil saturation in the three sand packs of the low-, medium-, and high-permeability layers are 48.8%, 24.9%, and 12.0%, respectively, and the total oil recovery is 54.5%. The CRF assistant steam flooding has a certain profile control ability, and the swept condition of the low-permeability layer is improved. The residual oil saturation decreased by 10.7%. The oil saturation of the medium-permeability layer has little change, and the oil recovery is also improved compared with steam flooding, which is increased by 7.9%. Although the effect of steam flooding with CX-5 is significantly better than that of steam flooding with CRF, the residual oil in the low-permeability zone is still as high as 22.7% at the end of the test. The ultimate recovery factor is further increased by 12%. Finally, the sweeping condition of steam flooding with PCS has been greatly improved. It not only improves the displacement effect of the low-permeability layer by 52.3% but also improves the displacement effect of the medium- and high-permeability layers, and the total oil recovery is 28.7% higher than that of steam flooding.

**Figure 12:**
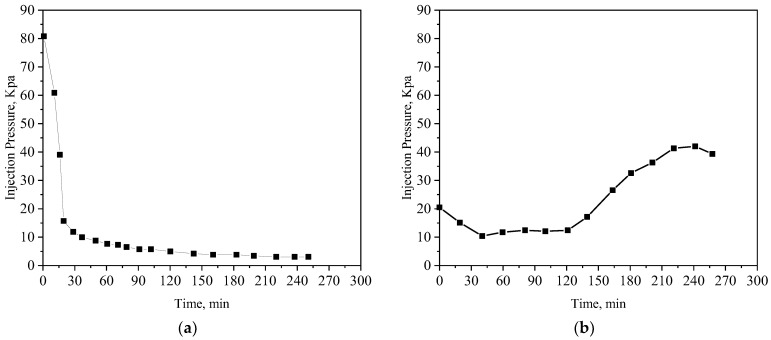
The pressure differential curve of sand-filled pipe core. (**a**) The steam flooding process, and (**b**) the PCS-assisted steam flooding process.

The differential pressure curves of steam flooding with and without PCS are shown in Figure 12. Consistent with the experimental results of the single tube flooding experiments, the injection–production pressure difference increases significantly after the injection of PCS, which has a strong effect on the expansion of the swept volume of the heterogeneous oil reservoir.

#### 3.5.3. Large-Size Rectangular Sand Filling Model Flooding Experiments

**Figure 13:**
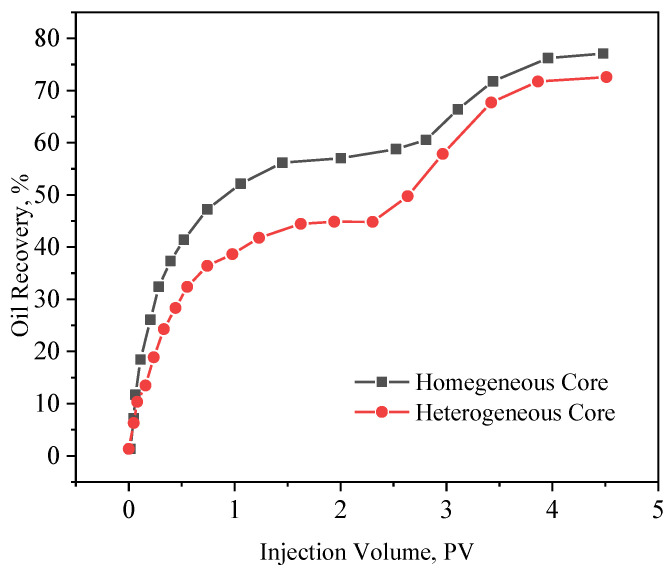
The curve of oil displacement efficiency and the number of injected PV.

During the experiment, steam flooding was carried out first. When the water cut of the produced fluid reached 98%, a 0.5 PV PCS solution was injected, and then continued steam flooding. When the water cut reached 98%, the experiment was terminated. Figure 13 shows the oil displacement efficiency curves of the homogeneous cores and heterogeneous cores. Figure 13 shows that for a large-sized homogeneous core, when the water cut reaches 98.0%, the oil displacement efficiency of conventional steam flooding is 57.53%. And the oil displacement efficiency is increased by 18.4% after the PCS flooding and second steam flooding. For the large-sized heterogeneous cores, when the water cut reaches 98.0%, the oil displacement efficiency of conventional steam flooding is 44.83%. After PCS flooding and the second steam flooding, the oil displacement efficiency increased by 27.86%. Comparing the results of the homogeneous and heterogeneous model flooding experiments, it can be found that the oil displacement efficiency is significantly improved when the water cut reaches 98.0% after steam flooding, indicating that the sweep efficiency of the homogeneous and heterogeneous cores has been improved. But PCS has a more obvious effect on the heterogeneous core, and its oil displacement efficiency increment is 9.46% higher than that of the homogeneous core.

## 4. Conclusions

In this paper, a high-temperature-resistant modified lignin, CRF, was developed and compounded with a foaming agent, CX-5, as a steam flooding profile control system (PCS). This paper systematically evaluates the foaming performance, resistance factor, and influencing factors of CX-5 and finally evaluates the enhanced oil recovery effect of the PCS-assisted steam flooding. The specific conclusions are as follows:

(1) CRF can be synthesized by sulfonation and the introduction of nonylphenol into the side chain of lignin molecules after oxidative chain scission. The FT-IR, UV-visible, and elemental analysis showed that CRF was successfully synthesized. CRF contains mostly syringyl groups, followed by guaiac groups, explaining its good temperature resistance. Low molecular weight, homogenization, and high activity are the directions for the development of lignin modification.
(2) CX-5 is a commercially modified anionic surfactant with a significantly increased molecular weight, giving it high-temperature resistance and interface activity. Its optimal concentration range is 0.3 wt%~0.5 wt%, the foaming volume currently is greater than 180 mL, and the foam half-life is greater than 400 s.
(3) The optimal formulation of PCS was determined by the resistance factors: 0.3 wt% CRF as an oil displacement agent + 0.5 wt% CX-5 as a foaming agent. The gas–liquid ratio is 1:2 to 2:1, and the formula system can withstand temperatures above 270 °C.
(4) The extent of reducing the residual oil saturation by PCS decreases with the increase in temperature. When the temperature is 200 °C, the oil displacement efficiencies of steam flooding with CRF, CX-5, and PCS are 5%, 2.1%, and 9% higher than that of steam flooding, respectively; and at 270 °C, the oil displacement efficiencies are 3.5%, 1.7%, and 7.9% higher than that of steam flooding, respectively. PCS can increase the injection–production pressure difference to more than eight times that of steam flooding, which can effectively expand the swept volume of steam.
(5) The three parallel tubes flooding experiment shows that the total oil recovery of PCS-assisted steam flooding increased by 28.7% based on steam flooding reaching 83.2%. PCS-assisted steam flooding can effectively improve the sweep efficiency of heterogeneous cores, which is the basic mechanism of EOR, resulting in a more than 9.46% EOR than the homogeneous cores. Our next research target is to release the microscopic pore throat flow rules and EOR mechanism of PCS-assisted steam flooding.

## Figures and Tables

**Table 1:** Experimental scheme of foam resistance factor test.

Number	CRF Concentration, wt%	CX-5 Concentration, wt%	Gas and Liquid Ratio	Temperature, °C	Pressure Difference, MPa	Permeability, D	Group
1	0.2–0.5	0.3	1:1	270	5.3	About 1.0	4
2	0.3	0.2–0.6	1:1	270	5.3	About 1.0	5
3	0.3	0.5	0:1–2:1	270	5.3	About 1.0	5
4	0.3	0.5	1:1	120–300	5.3	About 1.0	5
5	0.3	0.5	1:1	270	5.3 and 8.0	About 1.0	2
6	0.3	0.5	1:1	270	5.3	1.0–15.0	4

**Table 2:** The characteristic absorption peak and assignment of infrared spectroscopy.

Wave Number, cm^−1^	Groups
3417/3436	Stretching vibration characteristic absorption peaks of hydroxyl O-H
2938, 2845	The characteristic absorption peak of methyl stretching vibration
1707	Characteristic absorption peaks of non-conjugated carbonyl groups
1603	Aromatic ring skeleton vibration absorption peak
1514	Aromatic ring C-H bending vibration
1400~1460	Characteristic absorption peaks of stretching vibration of C-H of methylene
1329	Vibrational absorption peaks of lilac-type benzene ring skeleton
1267	Guaiac wood ring (C-O)
1219	C-O stretching vibration of the syringyl ring
1124	Syringyl group
1061	-SO_3_- antisymmetric and symmetric bond stretching vibration peaks coincide
1032	Secondary alcohol or ether (C-H bending vibration)
835	Out-of-plane C-H vibrations of aromatic rings at the 2, 5, and 6 positions (guaiacol type)

**Table 3:** The element analysis results of lignin.

Elements	C	H	O	S	C/H
Contents, %	55.55	6.28	31.93	6.24	8.81

**Table 4:** The influence of different gas–liquid ratios on the resistance coefficient.

Gas–Liquid Ratio	Permeability, D	Basic Pressure, Mpa	Work Pressure, MPa	Resistance Factor
0:1	1.05	0.0104	0.0718	6.9
1:2	1.07	0.0107	0.0813	7.0
1:1	1.07	0.0106	0.0901	8.5
2:1	1.09	0.0112	0.0833	7.5
3:2	1.06	0.0105	0.0704	6.7

**Table 5:** The influence of temperature on the resistance factor.

Temperature, °C	Permeability, D	Basic Pressure, MPa	Work Pressure, MPa	Resistance Factor
120	1.11	0.0099	0.4990	50.4
150	1.13	0.0103	0.2029	19.7
200	1.08	0.0096	0.1123	11.7
270	1.07	0.0095	0.0796	8.4
300	1.09	0.0097	0.0563	5.8

**Table 6:** The influence of return pressure on the resistance factor.

Back Pressure, MPa	Permeability, D	Basic Pressure, Mpa	Work Pressure, MPa	Resistance Factor
5.3	1.07	0.0095	0.0796	8.4
8	1.09	0.0103	0.1079	10.5

**Table 7:** The influence of permeability on the resistance factor.

Permeability, D	Basic Pressure, Mpa	Work Pressure, MPa	Resistance Factor
1.07	0.0105	0.0796	8.4
4.38	0.0082	0.0812	9.9
9.80	0.0063	0.0706	11.2
15.2	0.0042	0.0487	11.6

## Data Availability

Data are contained within the article.

## References

[1] Lyu X., Liu H., Pang Z., Sun Z. (2018). Visualized study of thermochemistry assisted steam flooding to improve oil recovery in heavy oil reservoir with glass micromodels. Fuel.

[2] Zhao D.W., Wang J., Gates I.D. (2013). Optimized solvent-aided steam-flooding strategy for recovery of thin heavy oil reservoirs. Fuel.

[3] Pang Z., Wang L., Yin F., Lyu X. (2021). Steam chamber expanding processes and bottom water invading characteristics during steam flooding in heavy oil reservoirs. Energy.

[4] Palatz (1989). Thermal Oil Recovery.

[5] Chen Y. (1996). Steam Injection Thermal Oil Recovery.

[6] Pang Z., Lyu X., Zhang F., Wu T., Gao Z., Geng Z., Luo C. (2018). The macroscopic and microscopic analysis on the performance of steam foams during thermal recovery in heavy oil reservoirs. Fuel.

[7] Shen P. (2006). Polymer Flooding Enhanced Oil Recovery Technology.

[8] Zhao F. (2008). Special Profile Control Agent for Thermal Recovery of Heavy Oil Wells.

[9] Wright T.R. (1980). Initial downhole steam generator tests completed. World Oil.

[10] Wright T.R. (1981). Comparative analysis of steam delivery cost for surface and downhole steam drive technologies. World Oil.

[11] Gao S., Li H., Yan Z., Pitts M.J., Surkalo H., Wyatt K. (1996). The alkaline/surfactant/polymer pilot performance of search, west central, Daqing oil field. SPE Reserv. Eng..

[12] Hu C. (1999). Heavy Oil Recovery Technology.

[13] Li Y., Chen X., Liu Z., Liu R., Liu W., Zhang H. (2021). Effects of molecular structure of polymeric surfactant on its physicochemical properties, percolation and enhanced oil recovery. J. Ind. Eng. Chem..

[14] Chen X., Li Y., Liu Z., Zhang J., Chen C., Ma M. (2020). Investigation on matching relationship and plugging mechanism of self-adaptive micro-gel (SMG) as a profile control and oil displacement agent. Powder Technol..

[15] Chen X., Li Y., Liu Z., Li X., Zhang J., Zhang H. (2020). Core-and pore-scale investigation on the migration and plugging of polymer microspheres in a heterogeneous porous media. J. Pet. Sci. Eng..

[16] Chen X., Li Y., Liu Z., Zhang J., Trivedi J., Li X. (2023). Experimental and theoretical investigation of the migration and plugging of the particle in porous media based on elastic properties. Fuel.

[17] Chen X., Li Y., Liu Z., Trivedi J., Tang Y., Sui M. (2023). Visualized investigation of the immiscible displacement: Influencing factors, improved method, and EOR effect. Fuel.

[18] Kong D., Gao J., Lian P., Zheng R., Zhu W., Xu Y. (2022). Characteristics of gas-oil contact and mobilization limit during gas-assisted gravity drainage process. Adv. Geo-Energy Res..

[19] Kong D., Gao Y., Sarma H., Li Y., Guo H., Zhu W. (2021). Experimental investigation of immiscible water-alternating-gas injection in ultra-high water-cut stage reservoir. Adv. Geo-Energy Res..

[20] Liu P., Zhang X., Wu Y., Li X. (2017). Enhanced oil recovery by air-foam flooding system in tight oil reservoirs: Study on the profile-controlling mechanisms. J. Pet. Sci. Eng..

[21] Lang L., Li H., Wang X., Liu N. (2020). Experimental study and field demonstration of air-foam flooding for heavy oil EOR. J. Pet. Sci. Eng..

[22] Chen X., Li Y., Sun X., Liu Z., Liu J., Liu S. (2023). Investigation of Polymer-Assisted CO_2_ Flooding to Enhance Oil Recovery in Low-Permeability Reservoirs. Polymers.

[23] Needham R.B. (1968). Plugging High Permeability Earth Strata. U.S. Patent.

[24] Paul G.W., Lake L.W., Pope G.A., Young G.B. A simplified predictive model for micellar-polymer flooding. Proceedings of the SPE California Regional Meeting.

[25] Scheren M. (1987). Parameter influencing porosity in sandstone a model for sandstone porosity prediction. AAPG Bull..

[26] Campbell T.C. (1982). The role of alkaline chemicals in the recovery of low-gravity crude oils. J. Pet. Technol..

[27] Duerksen J.H., Hsueh L. (1983). Steam distillation of crude oil. SPE J..

[28] Gorgarty W.B. (1967). Mobility Control with polymer Solutions. SPE J..

[29] Smith J.E. Quantitative Evaluation of Polyacrylamide Crosslinked Gels for Enhanced Oil Recovery. Proceedings of the International ACS Symposium.

[30] Clampitt R.L. (1976). Selective Plugging of Formations with Foam. U.S. Patent.

[31] Dilgren R.E. (1978). Stram-Channel-Expand Steam Foam Drive. U.S. Patent.

[32] Dilgren R.E., Owens K.B. (1979). Hot Water Foam Oil Production Process. U.S. Patent.

[33] Borchard J.K., Bright D.B., Dickson M.K., Wellington S.L. Surfactants for CO_2_ Foam Flooding. Proceedings of the SPE14394.

[34] Ettinger R.A., Radke C.J. (1992). Influence of texture oil steady foam flow in Berea sandstone. SPE Reserv. Eng..

[35] Schramm L.L., Turta A.T., Novosad J.J. (1993). Microvisual and core flood studies of foam Interactions with a light crude oil. SPE Reserv. Eng..

[36] Majeed T., Kamal M.S., Zhou X., Solling T. (2021). A review on foam stabilizers for enhanced oil recovery. Energy Fuels.

[37] Zhang Y., Liu Q., Ye H., Yang L., Luo D., Peng B. (2021). Nanoparticles as foam stabilizer: Mechanism, control parameters and application in foam flooding for enhanced oil recovery. J. Pet. Sci. Eng..

[38] Bhatt S., Saraf S., Bera A. (2023). Perspectives of Foam Generation Techniques and Future Directions of Nanoparticle-Stabilized CO_2_ Foam for Enhanced Oil Recovery. Energy Fuels.

[39] Abdelaal A., Gajbhiye R., Al-Shehri D. (2020). Mixed CO_2_/N_2_ foam for EOR as a novel solution for supercritical CO_2_ foam challenges in sandstone reservoirs. ACS Omega.

[40] Richardson E.A., Scheuerman R.F. (1973). Plugging Solution Precipitation Time Control.

[41] Wang M. (1994). Thermal oil recovery and enhanced oil recovery. Oil Gas Recovery Technol..

[42] Dong L., Li Y., Wen J., Gao W., Tian Y., Deng Q., Liu Z. (2022). Functional characteristics and dominant enhanced oil recovery mechanism of polymeric surfactant. J. Mol. Liq..

